# Expression of the selenoprotein S (*SELS*) gene in subcutaneous adipose tissue and *SELS* genotype are associated with metabolic risk factors

**DOI:** 10.1016/j.metabol.2010.05.011

**Published:** 2011-01

**Authors:** Maja Olsson, Bob Olsson, Peter Jacobson, Dag S. Thelle, Johan Björkegren, Andrew Walley, Philippe Froguel, Lena M.S. Carlsson, Kajsa Sjöholm

**Affiliations:** a Sahlgrenska Center for Cardiovascular and Metabolic Research, Department of Molecular and Clinical Medicine, Institute of Medicine, The Sahlgrenska Academy, University of Gothenburg, S-413 45 Gothenburg, Sweden; b Department of Biostatistics, Institute of Basic Medical Sciences, University of Oslo, N-0317 Oslo, Norway; c Department of Public Health and Community Medicine, Institute of Medicine, The Sahlgrenska Academy, University of Gothenburg, S-405 30 Gothenburg, Sweden; d The Computational Medicine Group, Atherosclerosis Research Unit, Department of Medicine, Karolinska Institutet, S-171 76 Stockholm, Sweden; e Section of Genomic Medicine, Hammersmith Hospital, Imperial College London, London W12 0NN, United Kingdom; f CNRS 8090-Institute of Biology, Pasteur Institute, Lille, France

## Abstract

The selenoprotein S (*SELS*) is a putative receptor for serum amyloid A, and recent studies have suggested that *SELS* may be a link between type 2 diabetes mellitus and inflammation. Genetic studies of *SELS* polymorphisms have revealed associations with circulating levels of inflammatory markers and hard end points of cardiovascular disease. In this study, we analyzed *SELS* expression in subcutaneous adipose tissue and *SELS* genotype in relation to metabolic risk factors. DNA microarray expression analysis was used to study the expression of *SELS* in lean and obese siblings from the Swedish Obese Subjects Sib Pair Study. TaqMan genotyping was used to analyze 3 polymorphisms, previously found to be associated with circulating levels of inflammatory markers, in the INTERGENE case-control study of myocardial infarction and unstable angina pectoris. Possible associations between *SELS* genotype and/or expression with anthropometry and measures of metabolic status were investigated. Real-time polymerase chain reaction was used to analyze the *SELS* expression in isolated human adipocytes incubated with insulin. In lean subjects, we found correlations between *SELS* gene expression in subcutaneous adipose tissue and measures of obesity (waist, *P* = .045; sagittal diameter, *P* = .031) and blood pressure (diastolic, *P* = .016; systolic *P* = .015); and in obese subjects, we found correlations with measures of obesity (body mass index, *P* = .03; sagittal diameter, *P* = .008) and glycemic control (homeostasis model assessment of insulin resistance, *P* = .011; insulin, *P* = .009) after adjusting for age and sex. The 5227GG genotype was associated with serum levels of insulin (*P* = .006) and homeostasis model assessment of insulin resistance (*P* = .007). The expression of *SELS* increased after insulin stimulation in isolated human adipocytes (*P* = .008). In this study, we found an association between both *SELS* gene expression in adipose tissue and *SELS* genotype with measures of glycemic control. In vitro studies demonstrated that the *SELS* gene is regulated by insulin in human subcutaneous adipocytes. This study further supports a role for *SELS* in the development of metabolic disease, especially in the context of insulin resistance.

## 1 Introduction

Selenoprotein S (*SELS*; also known as *SEPS1*, *AD-015*, or *SELENOS)* was recently described as an endoplasmic reticulum (ER) and plasma membrane-located selenoprotein involved in the physiologic adaptation to ER stress [1-4]. The *SELS* gene is known to be expressed in a wide variety of tissues and cell types, including tissues important for glycemic control such as adipose tissue, muscle, and liver [1,4-6]. In omental adipose tissue from diabetic subjects, gene expression of *SELS* is increased compared with that in nondiabetic controls; and in both groups, *SELS* expression correlates with homeostasis model assessment of insulin resistance (HOMA-IR) [7]. In HepG2 cells, *SELS* expression is inhibited by glucose; and *SELS* has been suggested to be involved in glucose homeostasis in an animal model of type 2 diabetes mellitus [6] and in human diabetic subjects [5]. Furthermore, in HepG2 and intestinal epithelial cells, proinflammatory cytokines activate the transcription of *SELS* [8,9]. The activation of *SELS* expression is influenced by a promoter polymorphism (rs28665122); and human studies have shown that *SELS* genotype is associated with circulating levels of proinflammatory cytokines, such as tumor necrosis factor–*α* (TNF-*α*) and interleukin-1*β* (IL-1*β*), suggesting that *SELS* plays a role in inflammation [10]. *SELS* is within a region of chromosome 15q26.3 that exhibits copy number variation in approximately 5% of individuals of European descent [11], but the frequency of this variation in the Swedish population is not known. It has also been shown that *SELS* is a putative receptor for the acute phase protein serum amyloid A (SAA) [6]. Consequently, it has been speculated that *SELS* may be a link between type 2 diabetes mellitus and inflammation. Recently, it was shown that *SELS* is secreted from hepatoma cells and that it associates with low-density lipoprotein (LDL) particles [12], suggesting a role also in lipid metabolism. Genetic studies of *SELS* polymorphisms in relation to cardiovascular disease have revealed that variation in the *SELS* locus is associated with coronary heart disease (CHD) and ischemic stroke in women [13].

Previously, genetic analyses of *SELS* in different cohorts have been performed [9,10,13,14]; and associations between *SELS* polymorphisms and inflammation [10] and hard end points in cardiovascular disease [13] have been found. In this study, we wanted to also include measures of glycemic control, lipid metabolism, and other risk factors as well as investigate possible associations with adipose tissue expression of *SELS* to further explore the role of *SELS* and the association with metabolic disease.

## 2 Materials and methods

### 2.1 Subjects and samples

#### 2.1.1 Ethics statement

Regional Ethical Review Boards approved the studies, and all participants gave written informed consent.

#### 2.1.2 The INTERGENE study

INTERGENE is a population-based research program assessing the INTERplay between GENEtic susceptibility and environmental factors for the risk of chronic diseases in western Sweden. The study population consists of randomly selected women and men, aged 25 to 74 years (at time of sampling), living in the Västra Götaland Region. The subjects included in this study are part of the INTERGENE case-control study, comprising 1236 (906 men and 330 women) well-characterized patients, of which 618 have suffered acute coronary symptoms (myocardial infarction or unstable angina pectoris) and 618 are matched healthy controls. The study is described in detail elsewhere [15] and at http://www.sahlgrenska.gu.se/intergene/. Body weight and height were measured to the nearest 0.1 kg and 1 cm, respectively. Waist circumference was measured at a level midway between the lower rib margin and iliac crest, and hip circumference was measured as the maximum perimeter over the buttocks. Blood pressure (BP) was measured twice in each person, after a 10-minute rest, with a validated (Gohara) automatic device (Omron 711 Automatic IS; Omron Healthcare, Vernon Hills, IL) while the subjects were in a supine position. Blood samples were collected after 4-hour fasting for immediate serum lipid (total cholesterol, high-density lipoprotein [HDL] cholesterol, LDL cholesterol, and triglycerides) and glucose analysis. Serum total cholesterol and triglyceride concentrations were determined with enzymatic assays. Serum HDL cholesterol concentrations were measured after dextran sulfate-magnesium precipitation of apolipoprotein B–containing lipoproteins. Serum glucose was analyzed with a hexokinase method (Roche Hitachi 917 and Roche ModularP; Roche, Basel, Switzerland). Measurements of serum insulin and high-sensitivity C-reactive protein (hs-CRP) were performed at the Sahlgrenska University Hospital.

#### 2.1.3 The Swedish Obese Subjects Sib Pair study

The Swedish Obese Subjects (SOS) Sib Pair study includes 154 nuclear families with body mass index (BMI)–discordant sibling pairs (BMI difference ≥10 kg/m^2^), resulting in a study population of 732 subjects. In this study, the most extreme siblings according to BMI were chosen in each family [16]. Sex discordant sib pairs were excluded, resulting in 78 pairs of sisters and 12 pairs of brothers. Measurements of anthropometry, fat mass (FM), fat-free mass (FFM), BP, fasting glucose, total cholesterol, triglycerides, HDL cholesterol, LDL cholesterol, serum insulin, serum C-peptide, and hs-CRP were performed at the Sahlgrenska University Hospital. Dual-energy x-ray absorptiometry was performed with LUNAR DPX-L (Scanexport Medical, Helsingborg, Sweden). The dual-energy x-ray absorptiometry generates a 3-compartment model consisting of FM, lean tissue mass, and bone mineral content. The FFM was calculated as lean tissue mass + bone mineral content.

### 2.2 Isolation and incubation of adipocytes

For isolation of adipocytes, subcutaneous adipose tissue was obtained from 5 women (BMI, 38 ± 17 kg/m^2^) and 4 men (BMI, 43 ± 10 kg/m^2^) [17]. One man and one woman received drug treatment for type 2 diabetes mellitus. Adipocytes were isolated from adipose tissue as previously described [18]. Subcutaneous adipocytes were diluted 10 times in modified Eagle medium and incubated with or without insulin (Sigma-Aldrich, St Louis, MO; 1 mU/mL, corresponding to 6 nmol/L) for 6 hours at 37°C in a gently shaking water bath. Total RNA was isolated from the adipocytes using the Lipid Tissue RNeasy Kit (Qiagen, Hilden, Germany).

### 2.3 Real-time polymerase chain reaction analysis

Reagents for real-time polymerase chain reaction (PCR) analysis of *SELS* and low-density lipoprotein receptor-related protein 10 (LRP10) (Assays-on-Demand, TaqMan Reverse Transcriptase reagents, and TaqMan GeneExpression Master mix) were purchased from Applied Biosystems (Foster City, CA), and conditions according to the manufacturer's protocol were used. Complementary DNA (cDNA) synthesis was performed in a total reaction volume of 20 *μ*L using 200 ng total RNA. cDNA corresponding to 10 ng RNA per reaction was used for real-time PCR amplification. Amplification and detection of specific products were performed using the ABI PRISM 7900HT Sequence Detection System (Applied Biosystems) using default cycle parameters. Working standards were prepared from a large pool of adipocyte RNA, and standard cDNA was synthesized in parallel with the sample cDNAs. A standard curve was plotted for each primer probe set with a serial dilution of the cDNA from the pooled adipose tissue RNA. Human LRP10 was used as reference to normalize the expression levels between samples [19].

### 2.4 Microarray analysis in the SOS Sib Pair study

Adipose tissue was obtained by needle aspirations in the paraumbilical area. Total RNA, cDNA, and hybridization (Human Genome U133 plus 2.0, Affymetrix, Santa Clara, CA) was performed as previously described [20-22]. Data were analyzed using robust multiarray average. *SELS* expression was analyzed using probe set 223209_s_at, and SAA expression was analyzed using probe set 214456_x_at.

### 2.5 Genotype analysis

Three *SELS* polymorphisms (Table 1), previously reported to be associated with serum levels of IL-1*β*, IL-6, and/or TNF-*α* [10], were selected for analysis. The C-105T polymorphism (rs28665122) is located within the *SELS* promoter, the C3705T polymorphism (rs4965814) is located in intron 5, and the A5227G polymorphism (rs4965373) is located within the 3′UTR of exon 6 [10]. Validated genotyping assays for the C3705T and the A5227G polymorphisms, a custom assay for the C-105T polymorphism (primer and probe sequences are available upon request), and TaqMan Universal PCR Master mix were purchased from Applied Biosystems and used according to the manufacturer's protocol. The ABI PRISM 7900HT Sequence Detection System (Applied Biosystems) was used for amplification and detection. To ensure genotyping quality control, negative controls were included in all genotyping plates. The DNA samples were transferred from 96-well stock plates to 384-well working plates using a Beckman Biomek FX (Beckman Coulter, CA) robotic system to minimize pipetting errors and to help eliminate sample plating errors. The genotyping success rate was 93%, which resulted in a total of 1150 subjects included in the full analysis and 535 matched case-control pairs available for paired analysis.

### 2.6 Statistics

Statistical analyses were performed using SPSS (version 16.0; SPSS, Chicago, IL). Values are given as mean ± SD unless stated otherwise. In the INTERGENE study, parameter difference for recessive and dominant models was assessed using independent samples *t* test. Multiple linear regression adjusted for diabetes status (subjects treated with insulin and/or other diabetic drug treatment at inclusion, with self-reported diabetes in questionnaire, or with serum glucose greater than 6.7 mmol/L), sex, and age were used to investigate possible associations between genotype and measured parameters. Only control subjects without lipid-lowering medication (eg, statin treatment) were included in the regression analysis of genotype effect on serum lipid levels. The genotype was coded to fit an additive model in the regression analyses. The Mantel-Haenszel test was used to test for association between genotype and risk for acute coronary symptoms. Significant deviation from the Hardy-Weinberg equilibrium was tested for each single nucleotide polymorphism (SNP) using a *χ*^2^ test. Linkage disequilibrium and haplotype frequencies were estimated using Haploview software version 4.1 [23]. PHASE 2.1 [24] was used to estimate the most likely haplotypes for each subject and to test if haplotype frequencies differed between cases and controls.

In the SOS Sib Pair study, correlation between *SELS* expression, SAA expression, and anthropometric and biochemical markers was performed using the Spearman rank correlation test. Partial correlation was used to control for sex, age, and BMI when appropriate. To obtain approximate normal distributions of expression data, microarray signals in the whole SOS Sib Pair study offspring cohort (n = 359) were transformed using Box-Cox power transformations. Subsequently, expression data were standardized to mean = 0 and variance = 1. Differences in gene expression between lean and obese siblings as well as *SELS* gene expression in isolated adipocytes incubated with or without insulin were assessed using a paired *t* test. A *P* value < .05 (2-sided) was considered statistically significant. This study is explorative in nature. Hence, we did not adjust for multiple testing.

## 3 Results

### 3.1 Association between *SELS* expression and metabolic risk factors in the SOS Sib Pair study

To further investigate *SELS* in relation with metabolic risk factors, we used the SOS Sib Pair study [16] to study *SELS* expression in relation to measures of glycemic control, blood lipids, anthropometry, and body composition. In all subjects, we found significant correlation only with serum levels of HDL cholesterol (*r* = −0.21, *P* = .005) when adjusting for sex, age, and BMI. In the lean subjects, we found significant correlations between *SELS* gene expression in subcutaneous adipose tissue and waist circumference, sagittal diameter, FFM, as well as systolic and diastolic BP after adjusting for age and sex (Table 2). In the obese subjects, we found significant correlations with BMI, sagittal diameter, serum levels of HDL cholesterol, triglycerides, insulin, and HOMA-IR after adjusting for age and sex (Table 2). No association was found between *SELS* and the expression of its putative ligand SAA after adjusting for age and sex in either group. Obese subjects had slightly but significantly higher *SELS* expression levels than lean subjects (*P* = .047; microarray signal, 45.1 ± 6.9 and 42.9 ± 7.3, respectively).

### 3.2 *SELS* expression in insulin-stimulated adipocytes

**Fig. 1:**
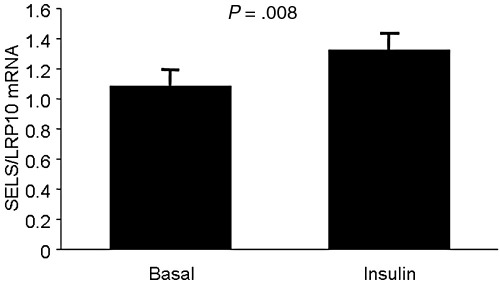
*SELS* expression in isolated human adipocytes stimulated with insulin in vitro (n = 9). LRP10 was used as a reference to normalize the expression levels between the samples. Values are presented as mean ± SEM.

To further investigate the link between glycemic control and *SELS* gene expression, we analyzed the effect of insulin stimulation on isolated subcutaneous adipocytes and found that the expression of *SELS* increased after insulin stimulation (Fig. 1, *P* = .008). Note that all subjects displayed increased expression after insulin exposure, except for one of the diabetic subjects in which the expression was unchanged. The increase was still significant after both type 2 diabetes mellitus subjects were excluded (*P* = .01).

### 3.3 Association between *SELS* genotype and cardiovascular and metabolic risk factors in the INTERGENE study

Using independent *t* tests, we found associations between the A5227G polymorphism and diastolic BP (*P* = .049), serum levels of HDL cholesterol (*P* = .049) and insulin (*P* = .006), and HOMA-IR (*P* = .007) for the dominant model for minor allele (AA + GA vs GG). For the recessive model for minor allele (AA vs AG + GG), an association was found between the A5227G polymorphism and diastolic BP (*P* = .019). For the C3705T and C-105T polymorphisms, no associations were found.

When the polymorphisms were included in a multiple linear regression model adjusting for diabetes status, sex, and age, the A5227G polymorphism was associated with diastolic BP (*P* = .008) and serum levels of insulin (*P* = .039) in all subjects. No association was found between the C-105T polymorphism and measured parameters, whereas the C3705T polymorphism was associated with serum levels of glucose (*P* = .043) and HOMA-IR (*P* = .034).

When analyzing the data using multiple linear regression in groups of cases and controls, we found associations only in the cases between the C-105T polymorphism with serum levels of insulin (*P* = .026) and HOMA-IR (*P* = .014), between the C3705T polymorphism with serum levels of insulin (*P* = .032) and HOMA-IR (*P* = .015), and between the A5227G polymorphism and serum levels of insulin (*P* = .038). In cases, we also found associations with diastolic BP (*P* = .048), BMI (*P* = .046), and waist-to-hip ratio (WHR) (*P* = .038) for the A5227G polymorphism, and an association with WHR (*P* = .036) for the C3705T polymorphism.

The associations between *SELS* genotype and BP in the whole cohort remained significant also when omitting subjects on antihypertensive treatment.

Using the Mantel-Haenszel test, we found that the 5227GG genotype was overrepresented among cases (odds ratio = 1.28; 95% confidence interval, 1.008 1.636; *P* = .043). No overrepresentation was found among cases for the other 2 polymorphisms.

The analyzed polymorphisms, A5227G, C3705T, and C-105T, were all in Hardy-Weinberg equilibrium and had minor allele frequencies comparable to those reported by Curran et al [10] (Table 1). Patient characteristics of the matched case-control pairs are shown in Table 3. Haplotype estimation identified 3 haplotypes with frequencies greater than 5% (CTG, 49%; CTA, 32%; and TCG, 13% in the order C-105T, C3705T, and A5227G); and together, these represented greater than 93% of haplotype diversity. No single haplotype was overrepresented among cases. See supplement for details on cohort and subject characteristics divided by genotype.

## 4 Discussion

In this study, we analyzed *SELS* expression in subcutaneous adipose tissue and *SELS* genotype in relation to metabolic risk factors. *SELS* genotype was associated with measures of glycemic control and diastolic BP. Furthermore, *SELS* gene expression was associated with measures of glycemic control and serum levels of HDL cholesterol in obese subjects, whereas *SELS* gene expression and BP were associated in lean subjects. *SELS* gene expression was also correlated with measures of obesity in both lean and obese subjects. We have also shown that *SELS* gene expression is stimulated by insulin in isolated human adipocytes.

*SELS* is expressed in 3 primary insulin target tissues: liver, muscle, and adipose tissue [6]. In an animal model of type 2 diabetes mellitus, the sand rat *Psammomys obesus*, it has been shown that *SELS* (tanis) is expressed in the liver in inverse proportion to circulating glucose and insulin levels [6]. It has also been shown that *SELS* expression is inhibited by glucose in cultured hepatocytes and that overexpression of *SELS* causes a reduction in glucose uptake in cultured hepatoma cells [1,6]. In humans, it has been shown that, in type 2 diabetes mellitus patients, *SELS* messenger RNA (mRNA) levels in subcutaneous adipose tissue tended to increase after insulin infusion, whereas no effect was seen in nondiabetic subjects [5]. In contrast, we found that *SELS* mRNA was up-regulated after insulin stimulation of isolated human subcutaneous adipocytes in nondiabetic subjects. Furthermore, we found that *SELS* mRNA was correlated with levels of insulin in obese subjects.

No correlation was seen between *SELS* mRNA levels in adipose tissue and glucose, insulin, or blood lipid levels in the study by Karlsson et al [5]. However, that study only included 10 type 2 diabetes mellitus subjects and 11 controls. In this study, we found positive correlations between *SELS* mRNA levels in subcutaneous adipose tissue and sagittal diameter in both lean (n = 90) and obese (n = 90) subjects after adjustment for sex and age. Furthermore, we found positive correlations between *SELS* mRNA levels in subcutaneous adipose tissue and waist circumference, FFM, and systolic and diastolic BP in the lean siblings and positive correlations with BMI, FM, serum levels of triglycerides and HDL cholesterol, insulin, and HOMA-IR in the obese siblings. Previous studies suggest a difference in *SELS* gene regulation depending on type 2 diabetes mellitus status in both subcutaneous [5] and omental adipose tissue [7]. Unfortunately, the cohort used for expression analysis in this study consists of relatively young subjects; and very few have been diagnosed with type 2 diabetes mellitus. Furthermore, no omental adipose tissue biopsies were obtained in this study*.*

It has been speculated that the interaction between the SAA1 protein and the putative receptor *SELS* [6] could be involved in the development of insulin resistance, and a recent study in rats showed that the expression level of *SELS* in liver was higher in diabetic rats compared with both healthy rats and diabetic rats treated with the insulin sensitizer rosiglitazone [25]. We show in this study that *SELS* expression is correlated with both insulin and HOMA-IR in the obese siblings, but no correlation was found in the lean group. In obese subjects, the levels of SAA in adipose tissue are higher than those in lean subjects, a fact that could possibly have effects on the ligand-receptor signaling even without large effects on *SELS* expression. However, further experiments using in vitro or in vivo models are needed to investigate this hypothesis.

*SELS*, and its interaction with SAA, has been proposed to be a mechanistic link between type 2 diabetes mellitus, inflammation, and cardiovascular disease [6]. Previous studies have shown that, in humans, both SAA and *SELS* adipose tissue expression are correlated with serum levels of SAA [5,26]. However, the relation between *SELS* and SAA expression in adipose tissue has previously not been studied. Although there was a weak correlation in the obese sibling group, the correlation was lost after adjusting for sex and age and was not present in the lean group, suggesting that *SELS* and SAA gene expression levels are not closely related in subcutaneous adipose tissue in humans.

Obesity and metabolic disease including atherosclerosis and insulin resistance are associated with modest but chronically elevated levels of circulating inflammatory markers [27,28]. It has previously been reported that 3 different *SELS* polymorphisms are associated with increased levels of circulating proinflammatory cytokines [10]. In this study, we analyzed these 3 polymorphisms in the context of the metabolic syndrome including risk factors such as obesity, measures of glycemic control, lipid metabolism, and BP. Using multiple regression analysis, we found associations between the C3705T polymorphism and measures of glycemic control (HOMA-IR and serum levels of glucose) and between the A5227G polymorphism and serum levels of insulin as well as diastolic BP. Using independent *t* tests, we found that the 5227GG genotype was associated with serum levels of insulin and HOMA-IR. Previous studies have suggested that the *SELS* gene is dysregulated in diabetes, and the data presented here indicate that the C3705T and/or the A5227G polymorphisms may play a role in this context. However, the C-105T polymorphism, which showed the strongest association with circulating proinflammatory cytokines [10] but no direct association with inflammatory bowel disease [9] or rheumatoid arthritis [14], was not associated with metabolic risk factors analyzed in this study.

In the study by Alanne et al [13], 2 polymorphisms, rs8025174 and rs7178239 (not analyzed in the INTERGENE study), showed significant associations with CHD and stroke, respectively. These associations were most pronounced in women. In this study, we found a weak association between risk of CHD and the A5227G polymorphism. No association with CHD was found for this polymorphism in the study by Alanne et al; but it was associated with serum levels of TNF-*α*, IL-1*β*, and IL-6 in the study by Curran et al [10]. It has to be considered that the number of subjects included in this study is about half of the number of subjects included in the study by Alanne et al [13]; and unfortunately, there were too few women in the cohort to perform the analysis split by sex. This may explain why the results for association with cardiovascular disease are different in the INTERGENE cohort.

In conclusion, we found an association between *SELS* gene expression and *SELS* genotype with measures of glycemic control. In vitro studies showed that the *SELS* gene is regulated by insulin in subcutaneous adipocytes. This study further supports a role for *SELS* in the development of metabolic disease, especially in the context of glucose homeostasis.

## Figures and Tables

**Table 1:** Information on the studied *SELS* polymorphisms

SNP ID^a^	Location	dbSNP ID	SNP sequence [minor/major allele]	Minor allele frequency (%)
C-105T	5′UTR	rs28665122	GTCGTGGTCC[T/C]GGCCAATCGC	14
C3705T	Intron 5	rs4965814	TACAGCTCAG[C/T]GTTTAAGGTC	18
A5227G	3′UTR	rs4965373	AGTAATAGTT[A/G]GAGGTTGTAA	33

a National Center for Biotechnology Information SNP data used as reference.

**Table 2:** Spearman correlation coefficients between *SELS* mRNA expression in adipose tissue and clinical parameters in the SOS Sib Pair study^a^

	Lean (n=90)			Obese (n=90)		
	Mean ± SD	*r*	*r* adj	Mean ± SD	*r*	*r* adj
BMI (kg/m^2^)	22.0 ± 1.7	0.19	0.15	37.7 ± 5.3	0.17	0.25⁎
Waist (cm)	77.0 ± 6.1	0.24⁎	0.23⁎	113.8 ± 12.4	0.04	0.17
WHR	0.80 ± 0.06	0.15	0.10	0.94 ± 0.07	−0.07	0.13
Sagittal diameter (cm)	18 ± 18	0.26⁎	0.24⁎	27 ± 37	0.15	0.31†
FM (kg)	17.6 ± 4.0	0.16	0.16	46.2 ± 9.5	0.30†	0.21
FFM (kg)	47.2 ± 7.5	0.32†	0.36‡	57.8 ± 8.2	−0.01	0.16
Systolic BP (mm Hg)	106.9 ± 9.5	0.27⁎	0.27⁎	121.3 ± 17.3	−0.04	−0.11
Diastolic BP (mm Hg)	65.3 ± 8.9	0.26⁎	0.27⁎	73.9 ± 11.4	0.11	0.19
Total cholesterol (mmol/L)	4.2 ± 0.9	0.04	0.02	4.6 ± 0.8	−0.16	−0.08
Triglyceride (mmol/L)	0.7 ± 0.4	0.15	0.12	1.3 ± 0.9	0.182	0.27⁎
HDL cholesterol (mmol/L)	1.4 ± 0.4	−0.20	−0.15	1.2 ± 0.3	−0.30†	−0.35†
LDL cholesterol (mmol/L)	2.5 ± 0.6	0.11	0.08	2.8 ± 0.6	−0.13	−0.07
Glucose (mmol/L)	4.7 ± 1.1	0.01	0.05	5.2 ± 1.0	−0.08	0.02
Insulin (mU/L)	5.6 ± 2.8	−0.08	−0.05	13.7 ± 9.7	0.20	0.30†
HOMA-IR	1.2 ± 0.7	−0.07	−0.04	3.3 ± 3.0	0.18	0.30⁎
C-peptide (mmol/L)	0.5 ± 0.2	0.21	0.21	0.9 ± 0.5	0.15	0.21
Hs-CRP (mg/L)	2.1 ± 5.5	−0.01	0.01	6.8 ± 7.4	0.06	0.05
SAA (microarray signal)	367 ± 130	0.01	0.13	448 ± 163	0.21⁎	0.10

a Spearman correlation (*r*) or partial correlation adjusted for sex and age (*r* adj) between *SELS* gene expression and clinical parameters was performed. Note that *r* adj was calculated for n = 79 and n = 73 in the lean and obese group, respectively.

⁎ *P* ≤ .05.

† *P* ≤ .01.

‡ *P* < .001.

**Table 3:** Characteristics of the subjects included in the paired *SELS* polymorphism analysis

	Cases (n=535)	Controls (n=535)
Diabetic (n)	125	51
Male/female (n)	383/152	383/152
BMI (kg/m^2^)	28.0 ± 4.3	26.8 ± 3.6
WHR	0.95 ± 0.08	0.91 ± 0.08
Systolic BP (mm Hg)	133.8 ± 21.1	142.6 ± 21.3
Diastolic BP (mm Hg)	81.8 ± 11.1	85.4 ± 10.5
Triglyceride (mmol/L)	1.7 ± 1.3	1.5 ± 0.8
Total cholesterol (mmol/L)	4.6 ± 1.1	5.8 ± 1.0
HDL cholesterol (mmol/L)	1.4 ± 0.4	1.6 ± 0.4
LDL cholesterol (mmol/L)	2.5 ± 0.9	3.5 ± 0.9
Glucose (mmol/L)	6.1 ± 2.3	5.5 ± 1.3
Insulin (mU/L)	14.1 ± 15.6	8.5 ± 6.7
Hs-CRP (mg/L)	4.7 ± 9.7	2.4 ± 4.0
