# Supplementary material for: Expression of the selenoprotein S (SELS) gene in subcutaneous adipose tissue and SELS genotype are associated with metabolic risk factors
Source: Metabolism. 2011 Jan;60(1-6):114–20. doi: 10.1016/j.metabol.2010.05.011 (PMC3004038; doi:10.1016/j.metabol.2010.05.011)
Supplement: Supplementary file 1 — Supplementary material [file mmc1.doc]

**Supplementary information on genotype versus cohort and subject characteristics in the INTERGENE study.**

|  | rs4965373 (A5227G) | | | | | | | | |
| --- | --- | --- | --- | --- | --- | --- | --- | --- | --- |
| Genotype | GG | | | AG | | | AA | | |
| n | 509 | | | 506 | | | 130 | | |
| Percent women | 26% | | | 29% | | | 27% | | |
| Percent cases | 57% | | | 49% | | | 57% | | |
| Percent T2D | 18% | | | 15% | | | 20% | | |
|  | Mean | ± | SD | Mean | ± | SD | Mean | ± | SD |
| age (year) | 61.0 | ± | 8.6 | 61.8 | ± | 8.2 | 62.2 | ± | 7.9 |
| BMI (kg/m^2^) | 27.6 | ± | 3.9 | 27.1 | ± | 4.0 | 27.3 | ± | 3.8 |
| WHR | 0.9 | ± | 0.1 | 0.9 | ± | 0.1 | 0.9 | ± | 0.1 |
| Systolic BP (mmHg) | 136.9 | ± | 21.3 | 139.0 | ± | 21.9 | 139.3 | ± | 21.6 |
| Diastolic BP (mmHg) | 82.7 | ± | 11.0 | 83.6 | ± | 11.4 | 85.5 | ± | 10.5 |
| Triglycerides (mmol/L) | 1.6 | ± | 0.9 | 1.6 | ± | 1.2 | 1.6 | ± | 1.0 |
| Total cholesterol (mmol/L) | 5.0 | ± | 1.2 | 5.3 | ± | 1.2 | 5.1 | ± | 1.2 |
| HDL cholesterol (mmol/L) | 1.5 | ± | 0.4 | 1.5 | ± | 0.4 | 1.5 | ± | 0.4 |
| LDL cholesterol (mmol/L) | 2.9 | ± | 1.0 | 3.1 | ± | 1.0 | 2.9 | ± | 1.0 |
| Fasting glucose (mmol/L) | 5.9 | ± | 2.2 | 5.7 | ± | 1.7 | 6.0 | ± | 2.0 |
| Insulin (mU/L) | 12.3 | ± | 12.9 | 11.1 | ± | 14.2 | 11.3 | ± | 11.8 |
| Hs-CRP (mg/L) | 4.2 | ± | 10.2 | 3.4 | ± | 6.6 | 3.6 | ± | 10.2 |

|  | rs4965814 (C3705T) | | | | | | | | |
| --- | --- | --- | --- | --- | --- | --- | --- | --- | --- |
|  | TT | | | CT | | | CC | | |
| n | 766 | | | 346 | | | 38 | | |
| Percent women | 26% | | | 29% | | | 37% | | |
| Percent cases | 52% | | | 55% | | | 55% | | |
| Percent T2D | 18% | | | 15% | | | 16% | | |
|  | Mean | ± | SD | Mean | ± | SD | Mean | ± | SD |
| age (year) | 61.5 | ± | 8.4 | 61.5 | ± | 8.3 | 61.9 | ± | 8.6 |
| BMI (kg/m^2^) | 27.2 | ± | 3.9 | 27.7 | ± | 4.0 | 26.9 | ± | 4.3 |
| WHR | 0.9 | ± | 0.1 | 0.9 | ± | 0.1 | 0.9 | ± | 0.1 |
| Systolic BP (mmHg) | 138.5 | ± | 21.4 | 137.2 | ± | 22.0 | 138.3 | ± | 22.6 |
| Diastolic BP (mmHg) | 83.4 | ± | 11.0 | 83.7 | ± | 11.6 | 81.6 | ± | 9.2 |
| Triglycerides (mmol/L) | 1.6 | ± | 1.1 | 1.6 | ± | 1.1 | 1.6 | ± | 1.1 |
| Total cholesterol (mmol/L) | 5.1 | ± | 1.2 | 5.2 | ± | 1.3 | 4.9 | ± | 1.1 |
| HDL cholesterol (mmol/L) | 1.5 | ± | 0.4 | 1.5 | ± | 0.5 | 1.5 | ± | 0.4 |
| LDL cholesterol (mmol/L) | 3.0 | ± | 1.0 | 3.0 | ± | 1.0 | 2.7 | ± | 1.0 |
| Fasting glucose (mmol/L) | 5.8 | ± | 1.9 | 5.9 | ± | 2.0 | 6.1 | ± | 2.9 |
| Insulin (mU/L) | 11.1 | ± | 11.5 | 12.7 | ± | 15.3 | 14.1 | ± | 25.2 |
| Hs-CRP (mg/L) | 3.7 | ± | 9.3 | 4.1 | ± | 7.7 | 3.5 | ± | 5.6 |

|  | rs28665122 (C-105T) | | | | | | | | |
| --- | --- | --- | --- | --- | --- | --- | --- | --- | --- |
|  | TT | | | CT | | | CC | | |
| n | 17 | | | 283 | | | 849 | | |
| Percent women | 29% | | | 30% | | | 27% | | |
| Percent cases | 47% | | | 54% | | | 53% | | |
| Percent T2D | 12% | | | 21% | | | 17% | | |
|  | Mean | ± | SD | Mean | ± | SD | Mean | ± | SD |
| age (year) | 65.6 | ± | 7.9 | 61.3 | ± | 8.3 | 61.5 | ± | 8.4 |
| BMI (kg/m^2^) | 26.8 | ± | 3.3 | 27.7 | ± | 4.2 | 27.2 | ± | 3.9 |
| WHR | 0.9 | ± | 0.1 | 0.9 | ± | 0.1 | 0.9 | ± | 0.1 |
| Systolic BP (mmHg) | 145.5 | ± | 22.6 | 138.6 | ± | 23.0 | 137.9 | ± | 21.0 |
| Diastolic BP (mmHg) | 80.9 | ± | 7.6 | 83.6 | ± | 11.7 | 83.4 | ± | 11.0 |
| Triglycerides (mmol/L) | 1.6 | ± | 1.1 | 1.5 | ± | 1.1 | 1.6 | ± | 1.0 |
| Total cholesterol (mmol/L) | 5.1 | ± | 1.0 | 5.2 | ± | 1.3 | 5.1 | ± | 1.2 |
| HDL cholesterol (mmol/L) | 1.4 | ± | 0.4 | 1.5 | ± | 0.5 | 1.5 | ± | 0.4 |
| LDL cholesterol (mmol/L) | 2.9 | ± | 1.0 | 3.0 | ± | 1.1 | 3.0 | ± | 1.0 |
| Fasting glucose (mmol/L) | 5.3 | ± | 0.5 | 6.0 | ± | 2.3 | 5.8 | ± | 1.8 |
| Insulin (mU/L) | 10.4 | ± | 7.0 | 13.4 | ± | 18.2 | 11.2 | ± | 11.4 |
| Hs-CRP (mg/L) | 3.8 | ± | 7.6 | 3.6 | ± | 5.8 | 3.9 | ± | 9.6 |
